# Influence of Composition and Spray-Drying Process Parameters on Carrier-Free DPI Properties and Behaviors in the Lung: A review

**DOI:** 10.3390/pharmaceutics12010055

**Published:** 2020-01-09

**Authors:** Anna Lechanteur, Brigitte Evrard

**Affiliations:** Laboratory of Pharmaceutical Technology and Biopharmacy, CIRM, University of Liege, 4000 Liège, Belgium; b.evrard@uliege.be

**Keywords:** lung, DPI, carrier-free, spray drying, péclet number

## Abstract

Although dry powder inhalers (DPIs) have attracted great interest compared to nebulizers and metered-dose inhalers (MDIs), drug deposition in the deep lung is still insufficient to enhance therapeutic activity. Indeed, it is estimated that only 10–15% of the drug reaches the deep lung while 20% of the drug is lost in the oropharyngeal sphere and 65% is not released from the carrier. The potentiality of the powders to disperse in the air during the patient’s inhalation, the aerosolization, should be optimized. To do so, new strategies, in addition to classical lactose-carrier, have emerged. The lung deposition of carrier-free particles, mainly produced by spray drying, is higher due to non-interparticulate forces between the carrier and drug, as well as better powder uniformity and aerosolization. Moreover, the association of two or three active ingredients within the same powder seems easier. This review is focused on a new type of carrier-free particles which are characterized by a sugar-based core encompassed by a corrugated shell layer produced by spray drying. All excipients used to produce such particles are dissected and their physico-chemical properties (Péclet number, glass transition temperature) are put in relation with the lung deposition ability of powders. The importance of spray-drying parameters on powders’ properties and behaviors is also evaluated. Special attention is given to the relation between the morphology (characterized by a corrugated surface) and lung deposition performance. The understanding of the closed relation between particle material composition and spray-drying process parameters, impacting the final powder properties, could help in the development of promising DPI systems suitable for local or systemic drug delivery.

## 1. Introduction

Pulmonary administration of drugs is gaining much importance in the latest research as it offers several advantages for the treatment of local or systemic diseases. Three major inhalation systems have been proposed for the aerosolization of drugs: nebulizers, metered-dose inhalers (MDIs) and dry powder inhalers (DPIs). Currently, DPIs, which deliver medication to the lungs in the form of a dry powder, represent the most promising system due to higher drug deposition in the deep lung. In addition, DPI inhalation systems do not use inhalation propellant gases detrimental to the environment and do not depend on the patient’s coordination during the inhalation process [1,2,3]. Besides local therapies developed against asthma or chronic obstructive pulmonary disease (COPD), lung administration using DPI has emerged as an alternative administration route for biopharmaceuticals like genes or peptides treating local and systemic diseases [4]. As mentioned by de Boer et al., the expectations for vaccination through the pulmonary route are currently high [5].

Drug efficacy is dependent on drug pulmonary deposition which depends on both the inhalation device performance and the aerosolization characteristics of the powder filled within it. Thus, when using DPIs for the aerosolization of drugs, the drug–device combination must be optimal to reach maximal drug efficacy. DPIs can be either “single-dose”, which use individual drug doses inside blisters or capsules, or “multi-dose” inhalers, which are basically composed of a reservoir filled with the drug powder. The design of new devices can intimately influence the lung delivery of drugs and should not be neglected [2,6,7]. However, this review is exclusively focused on powder particle engineering.

**Figure 1:**
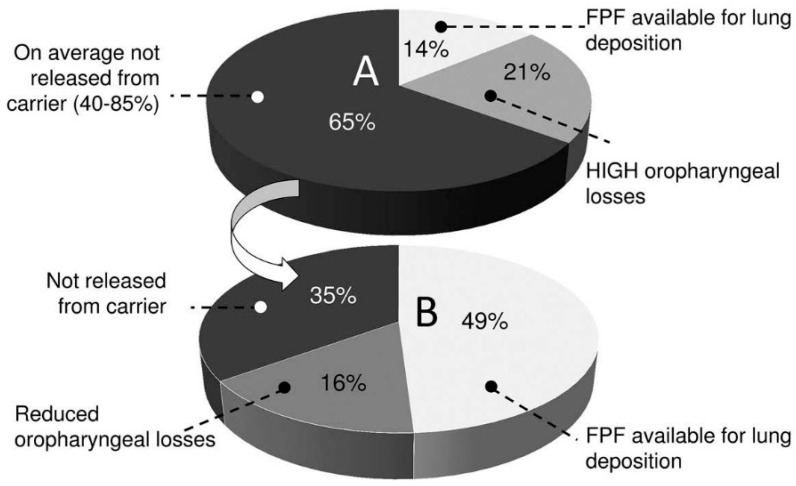
Fine particle fraction (FPF) of the dose delivered from dry powder inhaler (DPI) containing adhesive mixtures (carrier–drug formulations). (**A**) Representation of the actual situation with an FPF available for lung deposition of 14%, high oropharyngeal losses (21%) and about 65% of the drug not released from the carrier. (**B**) Representation of the ideal situation that would be 49% of FPF, lower oropharyngeal drug losses (16%) and lower drug rates (35%) that are not released from the carrier. Reproduced from [5], which is licensed under the terms of the Creative Commons Attribution-NonCommercial-NoDerivatives License (http://creativecommons.org/licenses/by-nc-nd/4.0/).

In order to reach the deep pulmonary drug deposition goal, DPI must ideally contain a powder made of the active pharmaceutical ingredients (API) coformulated with a range of excipients, which are chosen based on their precise functions within the powder, leading to optimal aerosolization performances and, consequently, high deposition into pulmonary regions. Traditionally, a sugar-based carrier (e.g., lactose, mannitol) is used in order to increase the poor aerosolization properties of API intended for lung delivery. Indeed, due to the low dosage of the API in some formulations and to the presence of cohesive fine particles which have high propensity to aggregate, pulmonary active drugs are almost never used alone but are combined with a carrier or “flow-aid”. Physical interparticulate forces between the carrier and API occur but are generally too intense, leading to poor delivery of drugs into the lung because of incomplete API–carrier dissociation [8]. Hence, the main challenge in drug aerosolization is to obtain the highest dose fraction deposition in the lower airways [9]. This criterion is experimentally defined as the fine particle fraction (FPF) and represents the proportion of emitted particles that have a lower particle size than the diameter of the upper airway, fixed at 5 µm [10]. As shown in Figure 1, the FPF for DPI containing adhesive carrier–drug powder mixtures is equal to only 14%, while 21% of the drug is lost in the oropharyngeal sphere and 65% are not released from the carrier. These results were obtained in vitro from thirteen commercial DPIs using a next generation impactor (NGI). It is obvious that to reach the desired situation in which at least 49% of the drug is available for lung deposition (Figure 1B), the drug dispersion in the air following the patient’s aspiration and the volatility of the powder should be increased [5].

Recently, a new strategy has emerged as very promising and innovative way to increase the pulmonary deposition of drugs. The development of carrier-free particles has been proposed to improve the uniformity and dispersibility of inhaled powders and consequently to increase the therapeutic activity of respirable drugs [9]. Different types of carrier-free particles are already on the market such as spheroids produced by the agglomeration of micronized budesonide (Pulmicort^®^) or porous particles called PulmoSpheres^®^ (Tobi Podhaler^®^ composed of tobramycin and PulmoSpheres^®^ platform) [2]. Moreover, different kinds of carrier-free particles are under study and investigation. For instance, Zhang et al. have very recently developed large porous microparticles loading budesonide using the single emulsion (O/W) solvent evaporation method with Poly(lactide-co-glycolide) and Poly(vinyl pyrrolidone) showing an FPF of ~21% [11]. Another new type of carrier-free particle, which is not well categorized yet, has emerged in recent years, and is characterized by a solid core surrounded by an outer layer. These particles are, most of the time, produced by spray drying [12], and have a low particle size (2–10 µm) with a wrinkled, rough surface. For a better understanding, these particles are named in this review as “composite-corrugated particles”.

**Figure 2:**
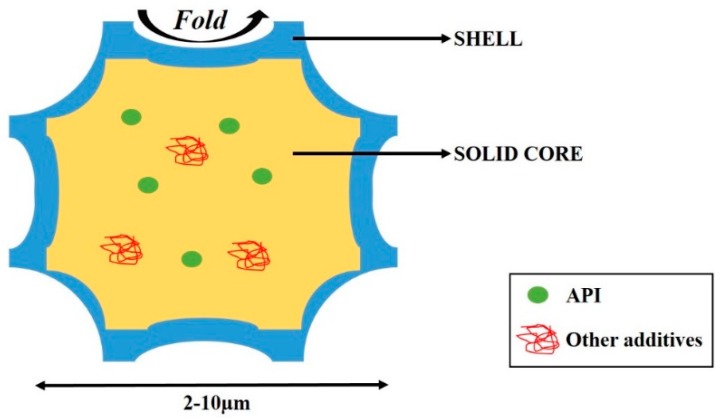
Representation of composite-corrugated particles produced by spray drying [13].

Figure 2 shows a schematic representation of these composite-corrugated particles made of a sugar-based core (yellow) encompassed by a shell layer (blue). By using this new strategy of powder production, there is no problem of blend uniformity or disaggregation since the API is entirely embedded in excipients. Understanding the importance of sugar properties and those of other excipients can lead to the development of particles containing different APIs that are effective for pulmonary delivery.

The spray-drying technique is suitable for the accurate engineering of inhalable particles since multiple parameters (Nozzle air flow, Inlet temperature, Solid content) can be modified during the production process which will impact, among other factors, the size, the morphology and the water content of microparticles [14]. Other techniques such as spray-freeze-drying and supercritical fluids could be used and are already reviewed by others [15,16].

In this review, we focus on the development of composite-corrugated particles produced by spray drying. All excipients used to produce composite particles will be listed in this work with special attention to their physico-chemical properties such as the glass transition temperature (Tg), the hygroscopicity and the Péclet number, all impacting the final powder behavior. Moreover, materials forming the core and/or the outer layer of microparticle powders are detailed and compared. Special attention is given to the spray-drying technique where the most important parameters, which influence the process yield and the powder morphology, are explained. Finally, the relation between particle properties such as surface texture and their behavior as well as DPI effectiveness will be dissected. Overall, this review gives an overview of the characteristics of all excipients used to develop composite-corrugated particles and their effectiveness to induce high aerosolization performance. The understanding of the closed relation between particle material composition and spray-drying process parameters, impacting final powder properties, could help in the development of promising DPI systems suitable for local or systemic drug delivery.

## 2. Excipients Forming Composite-Corrugated Particles

Spray drying is often used to produce dry powder microparticles. Indeed, this technique consists of the atomization of a solution or suspension through a nozzle which produces droplets. These droplets are rapidly dried due to high temperature inside the spray dryer. During the drying process, many excipients become amorphous, being in an unstable state, which leads to the production of a hygroscopic powder sensitive to agglomeration [17]. The physical stability of the powder must thus be optimized by using protective excipients forming a hydrophobic shell around the particle in order to obtain free-flowing powders. All excipients available for powder production are described in this section.

### 2.1. Excipients Forming the Core Compartment of Powder

The core of powder particles is, most of the time, made of carbohydrates such as oligosaccharide and polysaccharide molecules which contain three to approximately 10 molecules of monosaccharide residues connected by glycosidic linkages [18]. The type and number of monosaccharides can vary, as well as the tridimensional conformation which can be linear or cyclic. These diverse major structural characteristics confer different functional properties to powders. Moreover, polyols such as mannitol which are usually used to produce coarse carriers [19] can also compose the core of the composite-corrugated particles [20,21]. Table 1 summarizes the polyol and all oligosaccharides/polysaccharides used by spray drying to produce the core of composite-corrugated particle powders. Many publications have demonstrated the interest in combining different carbohydrates and this aspect will be discussed in Section 2.3.

#### 2.1.1. Influence of Molecular Weight, Hygroscopicity, and Glass Transition Temperature of Sugars on Powder Flowing Abilities

One major characteristic of sugars to take into consideration for spray-drying processes is the glass transition temperature (Tg), which is defined as the temperature at which an amorphous system changes from the brittle glassy state to a viscous rubbery state. Tg of carbohydrates varies according to their molecular weight. Therefore, the Tg of monosaccharides such as glucose, galactose, and fructose (Mw of 180.15 g/mol) are, respectively, 30, 31, and 10 °C [35]. The Tg of mannitol (Mw of 182.2 g/mol) is hard to measure since it is very low and consequently the tendency to crystallize is very strong [36]. On the contrary, trehalose (Mw of 342.3 g/mol), having a higher Tg (±100 °C), has often been reported to stabilize dry active molecules due to its amorphous state at room temperature [37].

The impact of Tg and the hygroscopicity of sugars during spray-drying processes on particle powder behavior is of great importance. This phenomenon has been reported in the literature on the drying of food products but has not yet been related in the context of DPI development. Zhang et al. explained recently that during the drying of materials bearing high sugar contents, there may be sticky residues on the inner chamber wall of the spray dryer. This behavior is related to the content of sugars with low molecular weights (glucose, galactose, lactose) having low Tg [38]. Indeed, sugars with low Tg are more hygroscopic, thus their moisture or water contents increase, which consequently decreases the flowing capacity of powders due to stickiness properties.

**Figure 3:**
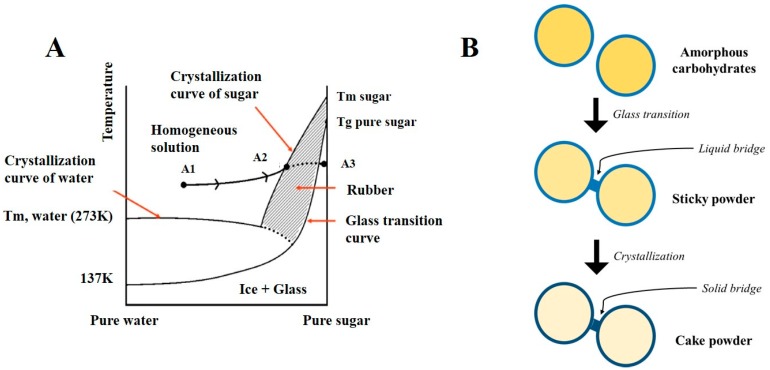
(**A**). Spray drying-state-diagram of a solution composed of sugar/water and a drug in a glassy matrix of sugar. (A1) Starting point composition. (A2) Point where the sugar solution (in the droplets) passes the crystallization curve of sugar. (A3) Spray-dried product in the amorphous glassy state. Reproduced from [39], which is an open access article distributed under the terms of the Creative Commons Attribution Noncommercial License (https://creativecommons.org/licenses/by-nc/2.0). (**B**). The stages of stickiness and caking in amorphous sugar particles. Adapted from [40], published by Bulletin de la Société Royale des Sciences de Liège (open access), 2017.

The understanding of the spray drying state diagram of a sugar solution in water can explain this sugar property (Figure 3A) [39]. The solution (A1) is atomized in the hot air flow during the spray-drying process, leading to the production of droplets. The evaporation of water induces an increase in sugar concentration within droplets (A1→A2) and sugars become solid either by crystallization or by amorphous precipitation, depending on the sugar used and its hygroscopicity/water content. For sugars bearing high molecular weight with a high Tg and low water content, if the evaporation is fast, the crystallization of the sugar cannot occur, thus the solution becomes a dry amorphous glassy powder instead of a sticky rubbery one (A2→A3). A sticky powder is a powder bearing cohesive or adhesive forces which stick to each other or to the wall of the equipment [40]. Hence, as shown in Figure 3B, for molecules bearing low molecular weight with high water contents, their stickiness is due to liquid bridges forming between particles upon glass transition, leading to a rubbery state. With further increasing temperatures, a caking of sugars occurs upon crystallization by the formation of solid bridges between particles. A high moisture content leads to solid bridge formation between individual particles, which promotes agglomeration.

**Figure 4:**
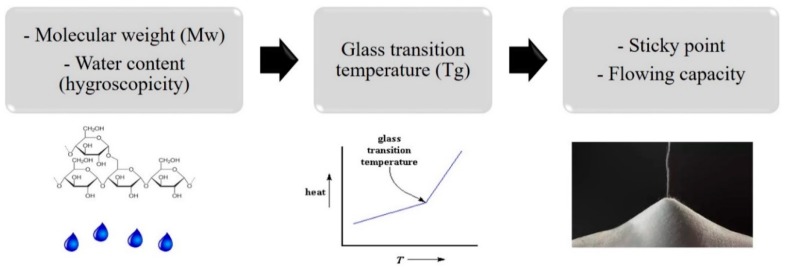
Influence of sugar properties on the flowing behavior of dry particles. Carbohydrates with a high molecular weight (Mw) and a low water content form sugars with a high glass transition temperature (Tg), which will produce a powder after drying with low stickiness and high tendency to flow freely.

Overall, the influence of sugar properties on the flowing behavior of dry particles is summarized in Figure 4. A sugar with high molecular weight and low hygroscopicity promotes a high glass transition temperature which will produce powder in an amorphous state with weak sticky behavior and therefore a higher flowing capacity. This also means that at room temperature, the powder is stable which positively influences the storage time of the product.

Gordon and Taylor proposed an equation to explain the relation between the water content and the Tg of the system [41]:

$$Tg=\frac{Tgm.Qs+kGT.Qw.Tgw}{Tgm+kGT.Tgw}$$

where Tg, Tgm, and Tgw are, respectively, the glass transition temperature of the system, the dry matrix and water. Qs and Qw are, respectively, the weight fraction solids and water in the system. kGT is the Gordon–Taylor coefficient.

If the Tg of a component is high (Tgm), the Tg of the system (Tg) is also high, which means that the amount of water that needs to be removed is lowered to reach the glassy state. Moreover, this equation explains that the solid content (Qs) impacts the water content that needs to be removed during the drying process. The influence of the solid content on powder particles will be investigated in Section 3 [41].

#### 2.1.2. Importance of Sugar’s Reducing Character on Active Ingredients’ Stability

Another property that has to be considered for sugars used in inhalation powder formulation is whether they have a reducing character or not. This character does not impact the morphology or behavior of the powder in the DPI but rather the stability of the API in the final product. Indeed, reducing sugars interact with amine groups (Maillard reaction) of API which, consequently, decrease their stability. Many APIs such as budesonide and formoterol, widely used in asthma therapeutic schemes, are involved, as well as peptides and proteins [19,42]. Almost all monosaccharides, some oligosaccharides like lactose, but no carbohydrates, considered to form carrier-free particles (Table 1), are reducing sugars.

### 2.2. Excipients Forming the Outer Shell of the Particle Powder

Besides sugars, other excipients can enter in inhalation powder composition, mainly to help the dispersibility of dry microparticles or to protect microparticles from moisture and therefore promote lung deposition.

Some materials used to form the outer shell of the particle are amino acids, among which the most well-known is L-leucine which has been reported to be present in many formulations to improve drug aerosolization performance [17,43]. Due to its high Péclet number (Pe), during the drying, L-leucine precipitates on the surface of droplets forming a hydrophobic layer which interferes with the diffusion of water and induces the formation of corrugated particles [44]. To be able to correlate the Pe number and excipients’ behavior, it is crucial to understand the mechanisms governing the drying of droplets.

#### 2.2.1. Drying Kinetics of a Droplet and Influence of the Péclet Number on Particle Powder Morphology

**Figure 5:**
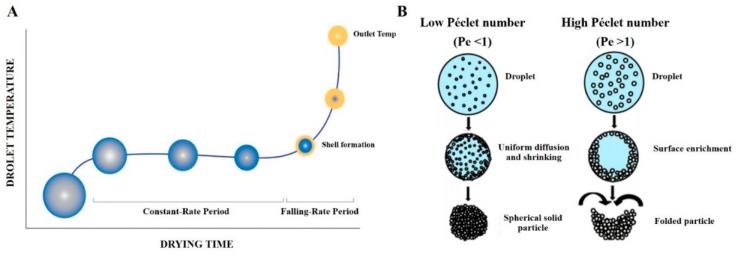
(**A**). Drying kinetics of a single droplet induced during the spray-drying process. Reproduced with permission from [49]. The graphic represents the evolution of the temperature inside a liquid droplet depending on the drying time. Two drying stages can be observed: the constant-rate period and the falling-rate period. (**B**). Schematic representation illustrating the different drying processes of droplets containing excipients having either a low Péclet number (Pe < 1, left panel) or a high Péclet number (Pe > 1, right panel). Inspired from [45], which is licensed under a Creative Commons Attribution (CC-BY) License.

The drying kinetics of a single droplet can be divided into two stages (Figure 5A) [45,46]. During the constant-rate drying (Stage 1), the solvent is evaporated constantly, the droplet temperature increases and the droplet size decreases. Step by step, the droplet shrinks due to solvent evaporation. Hence, molecules move within the droplet according to diffusion rates. Then, the solidification occurs, forming a shell or a skin layer around the droplet. At this stage (called falling-rate period), the shrinkage is very limited, whereas collapse of the shell may occur depending on the excipients used. Due to the solidification, the diffusion of solvent is poor and solid microparticles are created.

The morphology of final microparticles is influenced by the Pe number of excipients which is a dimension number calculated as the ratio between solvent evaporation rate κ (also called convection) and the diffusion rate of individual solute molecule *D* [45,47].

$$Pe=\frac{\kappa }{\theta D}$$

Zhang et al. have also explained which parameters influence the evaporation rate [11]. The latter is variable depending on the spray-drying parameters and solution/suspension composition. Indeed, a higher inlet temperature of the spray dryer induces a faster evaporation rate, thus a higher Pe number. This aspect is described in the next section. Furthermore, the solubility and the concentration of excipients and API modify the evaporation rate. More hydrophobic excipients tend to have a higher Pe number due to higher evaporation rates and consequently they enrich the particle surface. The opposite occurs for hydrophilic excipients. Mathematical explanations about this phenomenon have been explained in depth by Chen et al. [48].

As shown in Figure 5B, with excipients characterized by a low Pe value (<1), the diffusion of materials inside the droplet is similar to the convection; therefore, the distribution of excipients is uniform, and spherical solid particles are produced. On the contrary, at a high Pe value (>1), the solvent evaporation rate within droplets is faster than the solute diffusion leading to the accumulation of the material at the surface of the droplet during the shrinkage. In other words, with high Pe, the diffusion of the solute to the center of the droplet is slower than the evaporation of the solvent, leading to folded or corrugated particles [17].

#### 2.2.2. Amino Acids

In 2013, Boraey et al. investigated the spray-drying parameters of L-leucine and budesonide from an ethanol/water (75/25 *v*/*v*) feed stock solution (Table 2) [50]. Indeed, multiple API-like corticosteroids have very low water solubility, thus excipients or co-solvents able to enhance their water solubility are required. It has been previously demonstrated that a feed stock solution of a solid content of at least 1% *w*/*v* is needed to produce powder particles bearing a size range able to target the low respiratory tract [47]. In this study, L-leucine, as a water-soluble and shell-forming excipient, and ethanol, as co-solvent, were used to increase the solubility of budesonide. Powder particles had an appropriate median mass aerodynamic diameter (MMAD) of 3.8 µm and the FPF reached 66%. Another study has demonstrated that the atomization of budesonide in ethanol alone produced a very cohesive powder which was therefore not compatible with an efficient lung deposition, confirming the importance of amino acids to enhance drug aerosolization performance [51]. The relation between the solid content and the particle size is explained in Section 3.

Rattanupatam et al. showed that the spray drying of L-leucine and budesonide (feed stock solution ethanol/water 20/80) produced a powder inducing an FPF of 49.4%, which was low compared to the physical mixture of this powder produced with mannitol (FPF 65%) [52]. These results could be related to high inter-particulate forces inducing high powder aggregation and low flowability capability.

**Figure 6:**
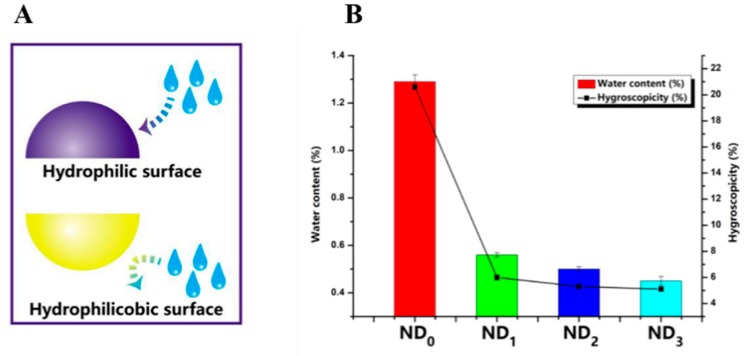
(**A**). Moisture absorption process of two different types of particles. The blue (above) surface is hydrophilic and thus sensitive to water absorption, whereas the yellow surface (below) is hydrophobic and protects the particle from ambient moisture. (**B**). Water content and hygroscopicity results of powder particles composed of different ratios of Netilmicin used as API and L-leucine. The mass ratio between Netilmicin and L-leucine for formulations ND0, ND1, ND2, and ND3 is, respectively, 100:1, 50:1, 30:1, and 10:1 (*n* = 3). Reproduced from [43], which is licensed under a Creative Commons Attribution-(CC BY 4.0) International License (http://creativecommons.org/licenses/by/4.0/).

In contrast to these examples working with low API doses, Cui et al. associated L-leucine to Netilmicin, a drug used at high doses in the context of bacterial infection of the lower respiratory tract [43]. Since the Netilmicin is highly hygroscopic, different L-leucine ratios were added (100:1; 50:1; 30:1; and 10:1 called respectively, ND0, ND1, ND2, and ND3) to form a hydrophobic layer around microparticles produced after spray drying and thus to reduce moisture absorption and particle aggregation (Figure 6A). Although powder particle diameters (d 0.5) were close to 3 µm and very similar, moisture contents were highly different among all above-cited formulations (Figure 6B). Formulation ND0 without L-leucine contained 1.3% of water and was highly hygroscopic compared to formulations ND1, ND2, and ND3 bearing lower water contents (~0.5%). Consequently, FPF for formulations ND0, ND1, ND2, and ND3 were 30%, 65%, 85%, and 48%, respectively. These results have been correlated to the morphology and shape of produced particles (Section 3).

Regarding the inhalation of Aztreonam for the treatment of local pulmonary infection, Yang et al. have recently compared the effects of three slightly water soluble amino acids (i.e., hydrophilic glycine, histidine containing an imidazol group and L-leucine) on particle characteristics when used in Aztreonam-combining formulations [53]. The spray drying of Aztreonam and amino acids (40/60) produced different powder particles in terms of density, morphology, and size. While glycine induced a very cohesive powder with a low production yield, a very big particle size (120 µm) and a very bad pulmonary deposition (FPF 0.29%), properties of powders produced with histidine and L-leucine were quite similar except their respective particle shape. Indeed, the histidine-based formulation promoted spherical particles, whereas microparticles made of L-leucine were winkled. The best FPF (61.7%) was observed for L-leucine-containing formulation and was related to the surface roughness.

Interestingly, Shetty et al. recently compared the physical stability of inhalation powders composed of ciprofloxacin and lactose, sucrose, trehalose, mannitol and L-leucine (50/50 *w*/*w*) [54]. They stored these powders at 20% and 50% relative humidity for 10 days and showed that, since L-leucine was the only excipient that did not absorb water, the L-leucine-containing formulation could induce a stable lung deposition (FPF stable at 75%). On the contrary, powder co-spray dried with lactose and ciprofloxacin and kept under relative humidity of 50% for 10 days induced a disappointing 0% FPF since the powder rapidly caked.

Trileucines have also been considered by Lechuda-Ballesteros et al. as interesting amino acids for powder formulations and have been associated with different API such as antibiotics (gentamicin and netilmicin) and anti-asthmatic drugs (albuterol and cromolyn) [55]. Regarding antibiotics, due to the high dosage of APIs, they have used the amino acid alone. Regarding anti-asthmatics, where only 2% of the formulation was the active drug (albuterol and cromolyn), raffinose was added to the formulation as an excipient in addition to 0%, 2%, and 15% of Trileucine. The authors showed a proportional increase in FPF depending on the percentage of added Trileucine. Interestingly, the authors have shown that all formulations produced without Trileucine (antibiotics alone or raffinose + albuterol/cromolyn) have a spherical morphology, whereas winkled particles were obtained regardless of the percentage of Trileucine added.

Finally, arginine was also used in the formulation of budesonide-containing powders produced by spray drying and compared to powders composed of only budesonide. Lu et al. have shown that 50% of powder composed only of budesonide remained stuck in the capsule and in the inhaler device, whereas the budesonide/arginine mixture (71/29 *w*/*w*%) provided a promising 85–90% of powder aerosolization. The role of arginine in promoting the dispersibility of the powder is not yet clear. The positive charges of arginine could generate an electrostatic repulsion between particles [56].

This section shows that amino acids can be used as the unique excipient to produce corrugated composite particles. However, two different kinds of API have been used: antibiotics and corticosteroids. With antibiotics (netilmicin, aztreonam), doses used in therapeutics are high, therefore amino acids were only added to enhance the aerosolization powder performance due to their high Pe and their ability to form a hydrophobic layer around the core of drug-composed particles. Concerning anti-asthmatic powders, some authors have used API and amino acids alone and have shown interesting aerosolization performances. However, high-speed capsule filling will not be easy due to the small amount of materials produced because of low therapeutic doses. In this sense, the use of other excipients is required to produce an adequate volume of free-flowing powder for inhalation powder industrialization.

### 2.3. Association of Materials to Produce Corrugated Composite Particles Produced by Spray Drying

In order to increase the volume of powder produced at industrial scale, carbohydrates can be used and sometimes associated with amino acids such as L-leucine to enhance the aerosolization performance of the inhaled powder. However, the exact amount of amino acids which should be added to carbohydrate excipient-API formulations has not yet been fully elucidated. While Chang et al. have shown that the addition of 10% L-leucine to herbal extract powder formulation was enough to protect powder particle from moisture [57], the amounts of L-leucine required in the context of pharmaceutical powder engineering have not yet been properly described in the literature. Table 2 shows an overview of sugar- and amino acid combinations used for pharmaceutical powder manufacturing by spray drying. The potential interest in using oligosaccharides and amino acids in inhalation powder engineering will be discussed in this paragraph.

#### 2.3.1. Association of Oligosaccharides and Amino Acids

To optimize the production of Trehalose/L-leucine powders for inhaled drug delivery, Focaroli et al. spray-dried different Trehalose/L-leucine solutions using different amino acid proportions, i.e., 70/30, 80/20, and 90/10 [17]. The authors concluded that a compromise must be found between the physical stability of the final powder and lung deposition performance. Indeed, the powder is better protected against environmental humidity with high L-leucine contents (20–30%), whereas higher deposition performance was obtained with powder containing lower amino acid quantities (10%).

In the context of systemic drug delivery of rivastigmine, microparticles were prepared by spray drying from solutions containing, in addition to the active ingredient, inulin combined with amounts of L-leucine ranging from 0% to 20%. Powder properties have been compared to the lactose-carrier formulation [32] and showed that, without L-leucine, API-inulin-powder particles were smooth, whereas folding in particles was observed proportionally to the amounts of L-leucine added to the combination. The yield during the spray-drying process was also higher when L-leucine was used because inulin alone is quite viscous and sticks to the wall of the spray-drying equipment. In this case, the highest FPF of 64.2% was obtained with the 20% L-leucine-containing formulation compared to 40.97% observed for lactose-carrier powder.

Interestingly, these data pinpoint the low number of articles published in the literature describing amino acid and sugar associations to develop carrier-free particles. This could be explained by the fact that some sugars are now proposed to play the same role as amino acids to form the outer shell layer of the microparticle.

#### 2.3.2. Association of Oligosaccharides

As shown in Table 2, few studies have combined two different oligosaccharides. For instance, Carrigy et al. have associated the quite hygroscopic trehalose with pullulan (higher Mw and Tg than trehalose). Despite the fact that the Pe number is not easy to determine for this combination, droplet chain experiments were performed and showed Pe numbers of 18 and 1 for pullulan and trehalose, respectively [33]. The combination of 10% pullulan and 90% trehalose showed winkled powder particles inducing an FPF of 40.1%. Moreover, the high Tg of pullulan (261 °C) can be advantageous to increase the powder stability compared to L-leucine or trileucine, having lower Tg (140 °C [17] and 104 °C [34], respectively).

##### Interest of Cyclodextrins in DPI Engineering

Some authors have investigated the use of cyclodextrins alone or in combination with other sugars to develop DPI. Cyclodextrins, which are cyclic oligosaccharides, have been widely used in drug formulations to enhance the aqueous solubility of API. Moreover, high interest in the formulation of powder for an administration through the inhalation route have been demonstrated. In the context of spray drying, cyclodextrins allow the preparation of stock feed solution having a high solid content. For instance, Dufour et al. produced inhalation powder from a HPβCD and budesonide (140/1) stock solution containing a solid content of 10% (*w*/*v*), which was extraordinarily high [29]. The biopharmaceutical properties of inhaled drugs should not only focus on the drug deposition rate. Indeed, once deposited into the conducting and respiratory zone of the lung, the administered powder should be dissolved and solubilized before being locally and/or systemically absorbed. We have to notice that there is actually a paradigm in the scientific community about the relation of the drug dissolution and its absorption related to its site of action. On the one hand, some research argues that a drug with a high dissolution speed and a high permeability will preferably have a systemic action, whereas prolonged drug dissolution will rather induce a local activity. On the other hand, it can also be true that a high dissolution speed and a high permeability promote an intense local absorption. This aspect was very well reviewed by Velaga et al. [6]. Due to the enhancement of water solubility, cyclodextrins/drug complexes can be more prone to induce a systemic action. HPβCD and DMβCD have been used with insulin, calcitonin, and hormones to increase their pulmonary absorption [60]. On the other hand, the mucoadhesive properties of cyclodextrins have already been described for buccal and nasal mucosa, leading to the sustained local activity of the drug [61]. Moreover, Dufour et al. demonstrated the pulmonary sustained delivery of budesonide from HPβCD using a cell-based model (Calu-3 cells barrier [62]), promoting a local anti-inflammatory action [29]. In this study, powder particles produced by spray drying had a wrinkled morphology and induced an FPF of 44%.

Another important point to take into consideration is the tendency that HPβCD has to form a shell layer around raffinose-core particles [26]. To demonstrate this claim, an experimental protocol was set up where HPβCD and raffinose were mixed in different proportions (0/100, 20/80, 30/70, 40/60, 60/40, 70/30, 80/20, and 100/0) and combined with budesonide (30/1 *w*/*w*); microparticles were produced by spray drying and physicochemical attributes were explored. Interestingly, the HPβCD/raffinose 60/40 formulation demonstrated the best flowability, higher air permeability, a lower tap density and low moisture absorption. Consequently, the FPF was 70.56% right after production and similar after a storage time of 10 days. The FPF of other formulations increased gradually from a 0/100 HPβCD/raffinose ratio to 60/40 and then decreased. The authors have also shown a correlation between the in vitro deposition results (obtained with the Next Generation Impactor, NGI) and pharmacokinetic profiles in rats. The behavior of HPβCD and raffinoe within the droplet during the drying has been suggested by authors. Indeed, they proposed that HPβCD forms a layer outside a raffinose core. This hypothesis could be explained by the fact that, during the drying, HPβCD concentration increased, leading to cyclodextrin aggregation. Due to the high hydrophilicity of raffinose (low Tg of 80 °C), its entry into the cyclodextrin cavity is prevented. Moreover, as explained in Section 2.2.1, the more hydrophobic component (HPβCD in this case) migrates preferably to the outer layer due to the high Pe number, whereas the more hydrophilic component (raffinose) easily migrates to the droplet’s center. In conclusion, due to the different physicochemical properties of raffinose and HPβCD, during the drying, the raffinose makes a solid core, whereas the cyclodextrin forms an outer layer around it. This explanation needs more evidence but is highly rational.

In sum, firstly, the choice of excipients is inevitably dictated by the drug properties. A high dosage of API (antibiotics) requires less excipients to be handled. On the contrary, a low quantity of API (anti-asthmatic drugs) needs different excipients to obtain a sufficient volume of produced powder with appropriate aerodynamic performance. Secondly, this section helps to understand how different excipients behave and are distributed within a particle of dry powder during the fast drying of a droplet. Oligosaccharides displaying a quite low molecular weight and low Tg (e.g., trehalose, raffinose) and which are consequently hygroscopic, may be combined with amino acids. Hence, L-leucine, having a high Peclet number, forms a hydrophobic layer around the core of sugar which prevents powder from water uptake and promotes good pulmonary deposition. Carbohydrates characterized by a high Pe number (cyclodextrins, pullulan) can perform likewise.

### 2.4. Other Additives

#### 2.4.1. Pore Forming Agents

As mentioned in the introduction, porous particles constitute another interesting strategy to develop DPI, having high aerosolization performance. We can consider why there would be interest in associating pore forming agents with excipients used to produce composite-corrugated particles.

Cyclodextrin metal-organic frameworks (CD-MOFs) have recently been described as a new type of particle which is the result of the combination between cyclodextrins and potassium ions [63]. The objective is to produce porous cubic powder particles supposed to be associated with a good lung deposition. However, until now, this technique has not proven to be highly successful since a formulation made of CD-MOF modified with cholesterol and carrying budesonide induced a poor 24.9% FPF.

Moreover, the use of volatile salts could be used as pore-forming agents as well. During the drying process, the salt is sublimated, and pores are created in the particles that are supposed to improve the dispersibility [64].

#### 2.4.2. Absorption Enhancers

Depending on the target delivery site of the powder, systemic absorption enhancers might be required. Some excipients have been proposed to promote the systemic absorption of drugs. For instance, in order to enhance the absorption of pDNA, Li et al. combined trehalose with three different absorption enhancers, namely dimethyl-β-cyclodextrin, sodium taurocholate and carnitine hydrochloride. The highest FPF (48.2%) was obtained with a formulation combining trehalose and dimethyl-β-cyclodextrin, proving a superior dispersibility. Moreover, trehalose/dimethyl-β-cyclodextrin powders showed an appropriate cell pDNA transfection efficiency on human lung epithelial carcinoma (A549) cells [65].

## 3. Spray-Drying Process Parameters

**Figure 7:**
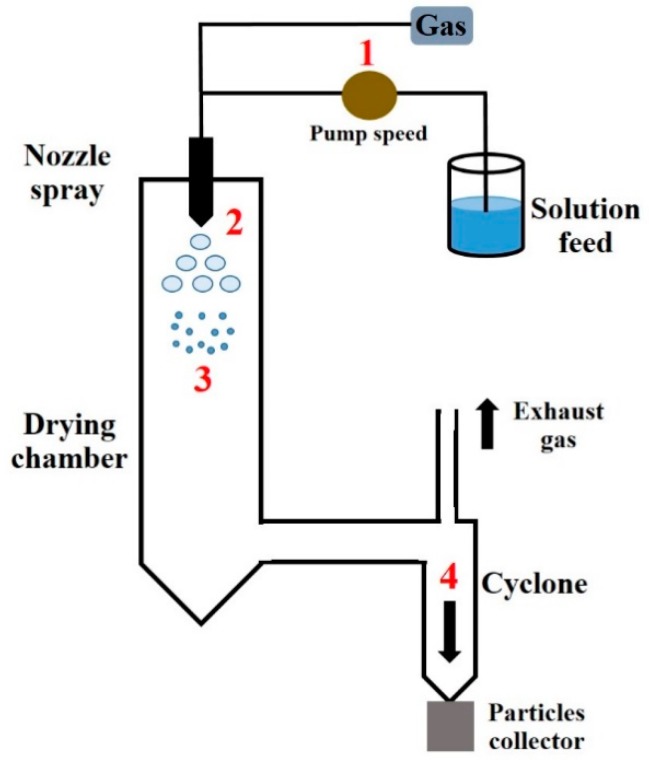
Four different steps of the spray-drying process. (**1**) The solution feed is pumped into the drying chamber according to the pump speed and atomized with the gas through a nozzle. (**2**) The drying of droplets occurs after the atomization. (**3**) Dry powder is formed in the drying chamber. (**4**) The separation of powder and air is induced by a cyclone in the bottom of the equipment and final powder is collected in a recipient.

The properties and ultimately the lung deposition of the final powder can be altered either by the formulation composition or by variation of the process factors. Spray drying is a technique based on the production of a dry powder following the atomization of a feed solution using a hot drying air stream. Four different steps (Figure 7) are described during the process: the atomization of the solution (1), the drying of droplets into drying air (2), the formation of dry powder (3) and the separation of powder and air (4) which is induced by a cyclone in the bottom of the device [66].

Multiple parameters can be adjusted during spray drying, which makes this technique very attractive to design particles with different size or shape characteristics. The process yield is highly important and is dependent on process parameters as well. Indeed, a sticky powder will adhere to the walls of the device, leading to a low yield. Since the production process is based on the atomization and on the evaporation of the solvent, the residual water content in the final product is consequently also dictated by the process. This section is focused on process parameters which impact the process yield and final powder characteristics. Some parameters, such as the inlet temperature (T°In), the airflow rate and the pump speed, are described to mainly impact particle powder characteristics. The influence of the main production parameters on particles properties are summarized in Table 3 and detailed hereafter. In addition to these adjustable settings, only the outlet temperature (T°Out) can be measured but informs about properties of the powder. The T°Out is the temperature measured in the bottom of the drying chamber and is the result of formulation composition (excipient + API) and of process parameters such as the feed solution rate and the T°In. A high T°Out promotes the formation of powder with low residual moisture. Therefore, the process yield will be high as well. Cabral-Marques et al. have shown that the FPF (%) is proportional to the T°Out. Indeed, they have shown that the best powder composed of beclomethasone diproprionate and γ-cyclodextrins and mixed with lactose induced the highest T°Out (38 °C) as well as the highest FPF of 50%.

### 3.1. Liquid Feed Concentration

As a reminder, the feed concentration, representing the solid particle fraction in the volume, can impact the particle size: a high feed concentration (>5% *m*/*v*) induces the production of droplets containing lower solvent amounts, thus leading to a higher solvent evaporation rate and therefore to the production of wrinkled particles (correlated to a high Pe number) [4]. In addition, the feed rate, dictated by the pump speed, has an impact on the particle size and the morphology. The feed rate corresponds to the transfer of feed solution into the nozzle per unit of time. It has been shown that increasing the pump speed (thus increasing the solution feed rate) results in larger particle size [17]. This can be explained by the fact that more fluid is provided, therefore the quantity of energy (given by the temperature and the pressure of air) per droplet is lower. This can also lead to particles bearing higher water contents due to insufficient drying [67].

### 3.2. Nozzle Air Pressure

Although there are different types of nozzle, such as ultrasonic or rotating, bi-fluid nozzles through which a liquid and a gas flow are most commonly used for pharmaceutical powder engineering [68]. The atomization gas flow influences the droplet size and density, ultimately affecting the properties of dry inhalation powder. Interestingly, the greatest influence on the particle size distribution is the airflow rate. Indeed, when the air pressure into the bi-fluid nozzle increases, the energy available to disperse the liquid feed and consequently break liquid droplets during atomization increases. Therefore, droplets are smaller as well as the final powder particles [17,69]. It has also been shown that a higher nozzle air pressure increases the production yield without any explanation to date [17].

### 3.3. Inlet Temperature

As reviewed by Singh et al., the T°In directly affects the heat and mass transfer phenomenon in the spray-drying droplet [68]. A high drying temperature leads to a faster drying because of more heat transfer into the drying droplet. Depending on the API and excipients used, the rapid formation of an outer layer on the droplet can occur, leading to porous particles or particles presenting corrugated surfaces. Mockedieck et al. have explained that T°In mainly influences the particle shape [21]. As previously discussed, the Pe number is influenced by the evaporation rate itself, impacted by the T°In. Indeed, a high T°In (> 120 °C) induces a fast solvent evaporation, thus a high Pe number leading to corrugated surfaces of particles. Moreover, it is obvious that a high T°In must lead to the formation of dry powder with low water content.

## 4. Aerosolization Performance Parameters

**Figure 8:**
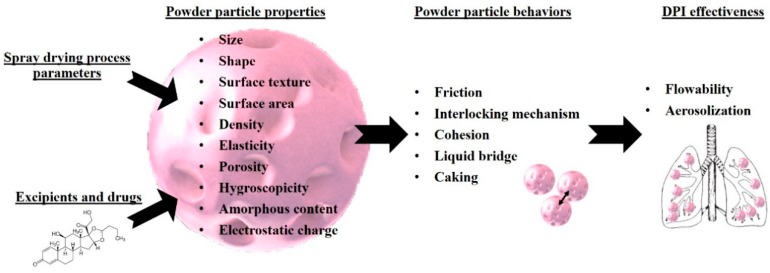
Powder particle properties impacting powder behaviors and consequently, the effectiveness of DPI.

The previous sections explain how the choice of excipients/API mixture and spray-drying parameters influence the resulting powder particle properties. This paragraph describes how such properties impact particle powder behaviors. Figure 8 summarizes all physicochemical properties conditioning powder behaviors and consequently, DPI effectiveness.

According to the equation of the aerodynamic diameter (*d_a_*), multiple parameters can influence the behavior of particles:

$$d_{a}=\sqrt[{6}]{\frac{\rho_{P}}{\rho_{0}}}\;\cdot \;\sqrt[{3}]{\frac{C_{0}}{\rho_{0}}}\;\cdot \;d_{0}$$

Hence, *d_a_* is the aerodynamic diameter, while *C*_0_, *ρ_P_*, *ρ*_0_ and *d*_0_ stand for the feed solution concentration, the particle density, the unit density and the initial droplet diameter, respectively.

A first observation indicates that the *d_a_* is dependent on the feed concentration stock solution (*C*_0_). As mentioned in Section 2.2.2, a feed stock solution of 1% has been described as optimal to produce powder particles displaying an appropriate size (1–5 µm). Regarding this equation, it is easy to understand that the more the *C*_0_ increases, the more the *d_a_* increases. Wang et al. have recently shown that the *d_a_* of a spray-dried formulation of trehalose increased from 5.98 µm to 15.50 µm when feed solutions of 1mg/mL and 30mg/mL were, respectively, atomized [70].

**Figure 9:**
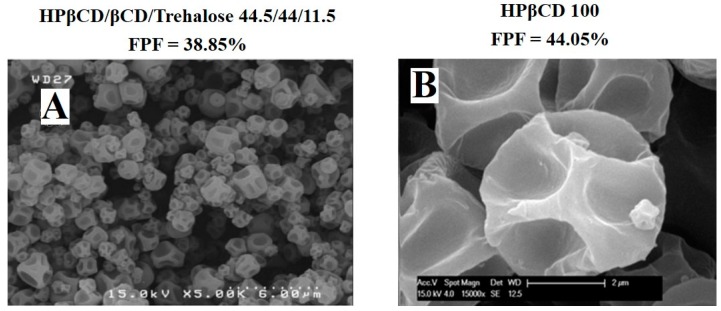
Scanning electron microscopy images of composite-corrugated particles showing rough surfaces. (**A**). Morphology 40), of microparticles composed of HPβCD, βCD, and trehalose (ratio 44.5/44/11.5), reproduced with permission from [25], published by Drug Development and Industrial Pharmacy, 2017. (**B**). Morphology of microparticles composed of 100% HPβCD reproduced with permission from [29], published by International Journal of Pharmaceutics, 2015.

Second, the weaker the particle density (*ρ_P_*), the smaller the *d_a_*. This explains why some authors have investigated the potential of large porous particles to produce efficient DPI. The incorporation of pores within powder particles decreases the density, characterized by the relationship between the weight (*m*) and the volume (*V*) ($\rho =\frac{m}{V}$). In the case of composite-corrugated particles, the decrease in density is induced by the roughness of particles. Indeed, as shown in Figure 9, many authors have shown that their particles had a folded or rough surface.

Due to folding, the distance between these particles is smaller, which prevents friction, interlocking forces and/or water bridge formations. Indeed, Zhao et al. proved that a spherical formulation composed only of raffinose displays physical properties which are completely different to corrugated particles made of HPβCD and raffinose (ratio 60/40). While diameters were similar among experimental groups (d0.5 = 4 µm), bulk density, aeration and permeability results were highly different, indicating a higher sensitivity to the airflow for corrugated particles [26].

Other authors who developed spray-dried particles with bovine serum albumin alone have studied in detail the degree of particle corrugation and surface morphology using scanning electron microscopy and atomic force microscopy. Due to modification of spray-drying processes, i.e., the feed concentration, the atomization rate and inlet temperature, different particles were produced. Hence, a reduction in the feed concentration induced the increase in surface corrugation. Moreover, using colloid probe microscopy, the adhesion forces between particles were evaluated and showed an inversed correlation between particle roughness and adhesion forces. Finally, a proportional correlation was found between the feed concentration (mg/mL), the particle roughness (nm) and the FPF (%) [71]. For a complete review of techniques used to study the microstructural characterization of powders such as pore structure or surface roughness, refer to Elsayed et al. [72].

**Figure 10:**
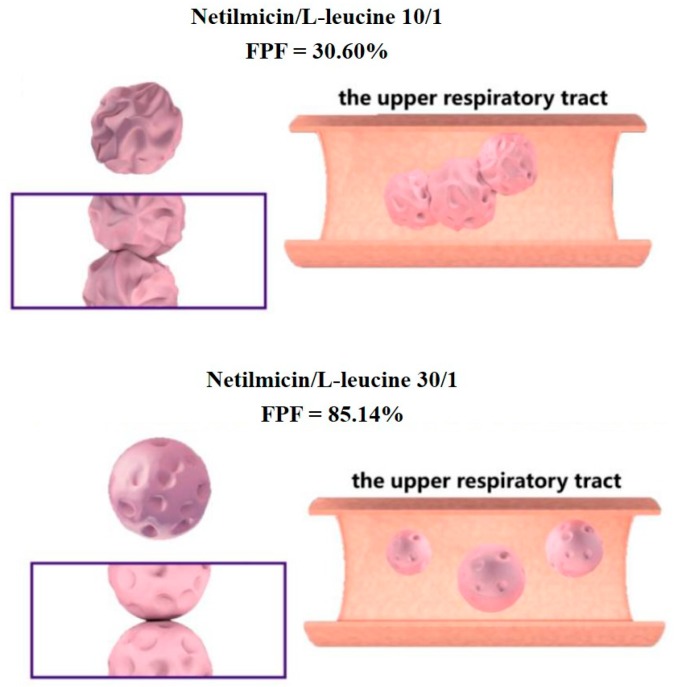
Schematic representation of the influence of a corrugated particle surface on the respiratory tract, reproduced from [43], which is licensed under a Creative Commons Attribution-(CC BY 4.0) International License (http://creativecommons.org/licenses/by/4.0/). Upper panel: Spray dried microparticles composed of Netilmicin and L-leucine (ratio 10/1) showed a highly rough surface and an FPF of 30.60%. Lower panel: Spray-dried microparticles composed of Netilmicin and L-leucine (ratio 30/1) showed a less rough surface and a high FPF of 85.14%.

Although the rough surface has shown great importance for respiratory deposition, Cui et al. have also warned about over-corrugated surfaces (Figure 10) [43]. Indeed, when surfaces are excessively folded, the particles become embedded in each other, inducing cohesion forces, agglomeration, and higher density, leading to poor aerosolization.

Another important parameter which is, most of the time, neglected is the electrostatic charges of powder particles. The contact between particles or with the device induces an exchange or donation of electrons during the production or inhalation process. The intensity of particle charges is dependent on many factors such as the particle size, morphology, surface, or water content [73]. To the best of our knowledge, this parameter is generally not considered when carrier-free particles are developed. One example for carrier particles was presented by Karner et al. They showed that the higher the triboelectric charges, the higher the FPF (~30%) [20]. However, in this study, mannitol was blended with salbutamol without spray drying, thus the size was 80 µm.

The importance of excipient characteristics has been largely discussed in previous sections which explained how this considerably impacts the hygroscopicity, the amorphous content or the morphology of final DPI.

Overall, it is clear that all parameters are closely related. While the atomization process influences the size of the particles, the size influences the aerodynamic diameter, itself influenced by the particle density or shape. Moreover, the choice of excipients determines the particle shape, the surface texture or the hygroscopicity. It seems very complicated to give numerical criteria that must be respected to produce DPI with high deposition performance even if trends can be found. Some physical parameters such as the size (0.5–5 µm), or technical factors such as solid content of the feed solution are well known to impact DPI performance. In this review, we have highlighted the importance of excipients’ characteristics in powder formulations in terms of Tg and hygroscopy, all parameters influencing the stickiness of the final powder. Moreover, it appears clearly that the morphology and surface texture of such small particles determine the aerosolization performance of powder particles.

## 5. Conclusions and Perspectives

This review describes the composition and the related lung deposition performance of carrier-free powder particles and more precisely of composite-corrugated particles. We have noticed that these particles are characterized by a solid sugar core surrounded by a corrugated outer layer with the drug directly embedded in it. The requirement of amino acids to form a hydrophobic layer around the core of the particle is not always necessary. Indeed, due to different physicochemical properties (molecular weight, Tg), the migration of material within the atomized droplet is different, leading to either a smooth or a wrinkled structure with a rough surface. The interest in cyclodextrins combined with other sugars has also been highlighted. A big improvement of these new formulations could be the fact that two or more active ingredients can be combined and consequently reach the same lung deposition. Moreover, with non-reducing sugars, having a high transition temperature (Tg), the amorphous state at room temperature is stable, which increases the product stability.

Regarding the morphology, the corrugated surface seems to be determinant in observing high deposition performance due to the reduction of interparticular forces, although more evidence is needed. Furthermore, additional studies are required to fully understand the importance of other particle properties such as the electrostatic charges. In the future, efforts should be made to ensure that a single excipient formulation can be used for different drugs, leading to the same pulmonary deposition.

## Figures and Tables

**Table 1:** Properties of polyol, oligosaccharides and polysaccharides used to produce the core of composite-corrugated particle powders by spray drying.

**Oligosaccharide**	**Type (Number of Monosaccharide)**	**Molecular Weight (g/mol)**	**Conformation**	**Tg (°C)**	**Reducing Sugar **	**Ref**
Mannitol	Polyol	182.2	Linear	/	No	[14,19,20]
Trehalose	Glucose (2)	342.3	Linear	97–106 [22,23]	No	[17,24,25]
Raffinose	Galactose (1), glucose (1), fructose (1)	504.4	Linear	80 [23]	No	[24,26]
β-CD	Glucopyranose (7)	1135.0	Cyclic	292 [27]	No	[25]
HP-β-CD	Glucopyranose (7)	1541.5	Cyclic	220 [28]	No	[25,26,29]
DM-β-CD	Glucopyranose (7)	1303.3	Cyclic	/	No	[30]
γ-CD	Glucopyranose (8)	1297.1	Cyclic	/	No	[30,31]
**Polysaccharide**	**Type (Number of Monosaccharide)**	**Molecular Weight (g/mol)**	**Conformation**	**Tg (°C)**	**Reducing Sugar**	**Ref**
Inulin	Fructose (23)	4 143.7	Linear	156	No	[32,33]
Pullulan	Maltotriose (n)	986.8	Linear	261	No	[34]

**Table 2:** Pharmaceutical powder engineering—oligosaccharide and amino acid combinations used to produce corrugated composite particles by spray drying and their relation to lung deposition efficacy (FPF).

Oligosaccharide(s)	Amino Acid	Optimal Ratio (%*w*/*w*) Oligosaccharides/Amino Acid (or If No Oligosaccharide, API/Amino acid)	API	Solvant (*v*/*v*)	FPF (%)	Solid Content (*w*/*v* %)	Ref
/	L-leucine	82.5/17.5	Budesonide	Ethanol/Water 75/25	66	0.7	[50]
/	L-leucine	1/50	Budesonide	Ethanol/water 20/80	49.4	1	[52]
/	L-leucine	97/3	Netilmicin	Water	85.1	3.3	[43]
/	Arginine	71/29	Budesonide	Water	61.6	1	[56]
/	Trileucine	85/15	-Gentamicine -Netilmicin	Ethanol/water 55/45	49.3 62.4	1 1	[55]
/	L-leucine	40/60	Aztreonam	Water	61.7	5	[53]
/	Histidine	40/60	Aztreonam	Water	51.4	5	[53]
/	Glycine	40/60	Aztreonam	Water	0.29	5	[53]
/	L-leucine	50/50	Ciprofloxacin	Water		1.6	[54]
Trehalose	L-leucine	90/10	/	Water	68.5	5	[17]
Inulin	L-leucine	80/20	Rivastigmine	Ethanol/water 30/70	64.2	1	[32]
Raffinose	Trileucine	84/16	Albuterol	Water	65	1	[55]
Lactose	L-leucine	80/20	Salbutamol	Water	78	2	[58]
HPβCD	/	/	Budesonide	Water	44	10	[29]
DM-β-CD	/	/	Budesonide	Water	67	-	[59]
Trehalose	/	/	Model protein (Lysozyme)	Ethanol/water 80/20%	62.3	1	[24]
Raffinose	/	/	Model protein (Lysozyme)	Ethanol/water 80/20%	50.1	1	[24]
Pullulan/Trehalose	/	10/90	/	Water	40	0.2	[34]
HPβCD/βCD/Trehalose	/	44.5/44/11.5	Antibody	Water	39	0.1	[25]
HPβCD/Raffinose	/	60/40	Budesonide	Water	70	2	[26]

**Table 3:** Influence of the main spray-drying parameters on particle powder properties.

Process Parameters	Particle Powder Properties
Liquid feed concentration	- Particle size
Feed rate	- Particle size - Particle shape - Water content
Nozzle air pressure	- Particle size - Production yield
Inlet temperature	- Particle shape - Water content

## Abbreviations

Tg	Glass transition temperature
API	Active pharmaceutical ingredients
Pe	Péclet number
MDIs	Metered-dose inhalers
DPI	Dry powder inhalers
COPD	Chronic obstructive pulmonary disease
FPF	Fine particle fraction
NGI	Next generation inhaler
MMDA	Median mass aerodynamic diameter
T°In	Inlet temperature
T°Out	Outlet temperature
